# Computation of Robust Minimal Intervention Sets in Multi-Valued Biological Regulatory Networks

**DOI:** 10.3389/fphys.2019.00241

**Published:** 2019-03-19

**Authors:** Hooman Sedghamiz, Matthew Morris, Darrell Whitley, Travis J. A. Craddock, Michael Pichichero, Gordon Broderick

**Affiliations:** ^1^Center for Clinical Systems Biology, Rochester General Hospital Research Institute, Rochester, NY, United States; ^2^School of Computer Science, Colorado State University, Fort Collins, CO, United States; ^3^Department of Psychology and Neuroscience, Nova Southeastern University, Fort Lauderdale, FL, United States; ^4^Department of Computer Science, Nova Southeastern University, Fort Lauderdale, FL, United States; ^5^Department of Clinical Immunology, Nova Southeastern University, Fort Lauderdale, FL, United States; ^6^Clinical Systems Biology Group, Institute for NeuroImmune Medicine, Nova Southeastern University, Fort Lauderdale, FL, United States; ^7^Center for Infectious Diseases and Immunology, Rochester General Hospital Research Institute, Rochester, NY, United States; ^8^Department of Biomedical Engineering, Rochester Institute of Technology, Rochester, NY, United States

**Keywords:** target identification, logical modeling, algorithms, signaling networks, experimental design, drug therapy

## Abstract

Enabled by rapid advances in computational sciences, *in silico* logical modeling of complex and large biological networks is more and more feasible making it an increasingly popular approach among biologists. Automated high-throughput, drug target identification is one of the primary goals of this *in silico* network biology. Targets identified in this way are then used to mine a library of drug chemical compounds in order to identify appropriate therapies. While identification of drug targets is exhaustively feasible on small networks, it remains computationally difficult on moderate and larger models. Moreover, there are several important constraints such as off-target effects, efficacy and safety that should be integrated into the identification of targets if the intention is translation to the clinical space. Here we introduce numerical constraints whereby efficacy is represented by efficiency in response and robustness of outcome. This paper introduces an algorithm that relies on a Constraint Satisfaction (CS) technique to efficiently compute the Minimal Intervention Sets (MIS) within a set of often complex clinical safety constraints with the aim of identifying the smallest least invasive set of targets pharmacologically accessible for therapy that most efficiently and reliably achieve the desired outcome.

## Introduction

Rapid advances in the computational sciences has enabled biologists to study complex biological phenomena using logical and mathematical techniques. Due to the close resemblance of biological networks to digital circuits (Abdi et al., 2008; Morris et al., 2010), logical modeling techniques have proven well-suited to the study of such phenomena. It has been shown that even complex biological behaviors such as cellular differentiation and multi-stationarity can be captured by logical analysis (Thomas and Kaufman, 2001a,b). Currently, reasonably large logical models can be identified by training to experimental data (e.g., phosphoproteomics) (Klarner et al., 2012; Guziolowski et al., 2013; Sedghamiz et al., 2017). Once a model is parameterized, biologists study its associated attractors (steady states) as well as the response dynamics of the system around and between these steady states to gain insight into illness onset and possible resistance to treatment. Often a particular attractor (or set of attractors and their associated basins) supports states that resemble a pathological phenotype. Therefore, one would like to understand how such intracellular, cellular, and organ system behaviors might be manipulated and redirected into an alternative phenotype (e.g., health). Intuitively, we want to identify an intervention strategy whereby a set of entities in the model are collectively modulated (down or up-regulated) in a way that the treatment-modified network preferentially accommodates states that evolve toward the healthy attractor. However, even with advances in drug compound design, direct, and indirect off-target effects can highly reduce the efficacy and safety of therapy. Part of this problem might be addressed by finding the minimal number of entities that must be targeted concurrently in order to force the transition from one cellular phenotype to the other. This is often denoted as finding the Minimal Intervention Sets (MIS).

The computation of MIS was first addressed (Karlebach and Shamir, 2010; Samaga et al., 2010; Verdicchio and Kim, 2011) only in moderate sized Boolean networks. Recently, efficient algorithms have been proposed in order to compute MIS for Boolean networks based on Branch and Bound (BB) (Garg et al., 2013) and Answer Set Programming (ASP) (Kaminski et al., 2013) techniques. However, both methods are only applicable to Boolean networks and support limited logical operators (e.g., AND, OR, etc.). Therefore, we still require a method that is broadly applicable to multi-valued models and a more expressive logical representation. With the exception of the algorithm provided by Garg et al. (2013), these other studies (e.g., Karlebach and Shamir, 2010; Samaga et al., 2010; Kaminski et al., 2013) do not formally consider possible indirect off-target effects during the computation of MIS patterns. Moreover, to our knowledge, none of the above-mentioned methods formally account for the robustness and efficiency of the state transition path associated with a MIS. We propose that ranking MIS patterns based on their efficiency and robustness supports screening of drug library compounds such that intervention solutions are more realistically translatable into practice. Importantly, Garg et al. (2013) also showed that the most interesting MIS patterns were observed when the initial state of the model was taken into account prior to the computation of the MIS, a result which is consistent with the trend toward personalized medicine especially as it applies to complex illness. In this manuscript, we propose an efficient Constraint Satisfaction (CS) based algorithm that addresses the MIS computation as a multi-objective problem where the objectives are to minimize the Complexity of the intervention, while maximizing its reliability or Robustness and the expediency of response or Efficiency. In addition, increased Safety is articulated as a reduction in the number of predicted off-target downstream effects. As explained earlier, due to the high number of constraints involved with identifying MIS such as Safety (e.g., downstream off-target effects), Robustness and Efficiency, constraint programming techniques seem to be well-suited. Our goal is to propose a MIS that:

- Maps a regulatory network to a desired goal or target steady state
- Maximizes the robustness and efficiency of the proposed intervention therapy
- Reports the downstream off-target effects and informs on its theoretical safety

Compared to previous contributions in this context, our work allows for the initial state of the network (e.g., attractor) to be considered as a constraint prior to the computation of MIS. Also, we formally consider the trajectory of transition from the initial state to the target state and find the most efficient (e.g., shortest) and robust (e.g., fewest deviations from the destination) path for this transition, using Monte-Carlo simulations under different levels of biological noise (Sedghamiz et al., 2018). Our proposed method is able to handle multi-valued and Boolean logic schemes, as well as the combination of both. Our framework consists of a preprocessing step where logic synthesis techniques are employed to simplify the dynamics associated with each entity with the help of Reduced Ordered Multivalued Decision Diagrams (ROMDDs). A more detailed description of these thresholds is given in section Simplification With ROMDDs. After simplifying the network, we employ One-hot encoding to convert a multi-valued network into an equivalent Boolean model. Finally, the simplified and converted model is analyzed based on three-valued Kleene's logic (Bergmann, 2008) to identify the MIS sets. The latter has been previously employed successfully for fault detection in electrical circuits (Abramovici et al., 1990). This paper is organized as follows. First, we review the multi-valued formalism. Then, we formally describe the necessary background in regards to the computation of intervention sets. Finally, we present criteria in order to rank the intervention sets and apply our algorithm on three biological networks namely; established benchmark models of the Hypothalamic Pituitary axis (HPA) (Sedghamiz et al., 2018) and T-helper differentiation (Garg et al., 2013) as well as a first novel model of immune signaling in young children in the first year of life who display a Low Vaccine Response (LVR) to two-thirds or more of their recommended routine immunizations.

## Materials and Methods

### Generalized Multi-Valued Formalism

**Figure 1:**
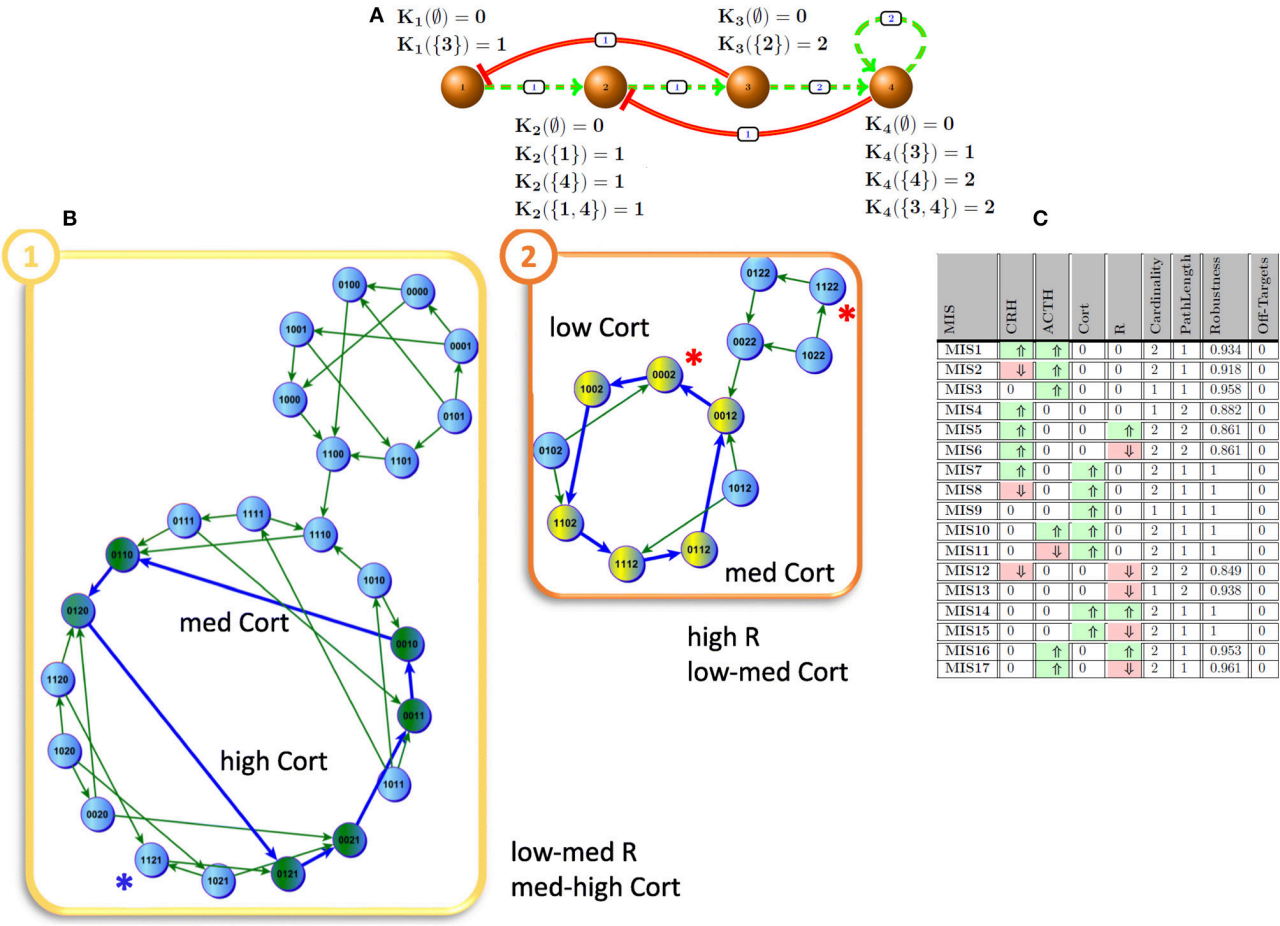
HPA axis. **(A)** HPA as a discrete network and its logical parameter sets; solid red and dashed green edges indicate inhibiting and promoting effects, respectively. The weights represent the expression threshold for above which the interaction becomes active. **(B)** The emergence of two cyclic attractors demonstrates the multi-stability of the model HPA axis as described by the 4 state variables CRH, ACTH, Cortisol, and R. In attractor 1, node 3 (Cortisol) oscillates between mid to high levels while in attractor 2 it oscillates between mid to low levels (Sedghamiz et al., 2018). **(C)** Intervention sets to induce mid-to-high node 3 (Cortisol) levels; ⇑ (light green), ⇓ (light red) indicate agonistic and antagonistic intervention effects, respectively.

In this study, we employ *Generalized Multi-valued Formalism* (GMF) that was proposed, developed and enhanced over decades by Kauffman (1969), Thomas et al. (1995), and Sedghamiz et al. (2017). In such a formalism, molecular signaling and regulatory actions are concentration dependent and the entities being modeled are allowed to assume more than binary values. In addition, a set of logical parameters (𝕂) are defined to explain the complex aggregate interaction of cofactors on a target. A basic example of stress hormone regulation by the hypothalamic-pituitary-adrenal (HPA) axis is described in GMF and shown in Figure 1A. In this example, the expression states of nodes *v*_1_ and *v*_2_ are denoted in binary values [e.g., low (0) and high (1)] while nodes *v*_3_ and *v*_4_ assume three states [e.g., 0 (low), 1 (medium), and 2 (high)]. By default, the number of states for each entity in the network is proportional to the number of entities they act upon (e.g., out-degrees). Each positive (negative) regulatory action in the graph is equipped with a threshold above which it becomes functional. For instance, for node *v*_1_, *K*_1_(∅) = 0 describes that this entity is deactivated once it has no activating regulator and *K*_1_({3}) = 1 indicates that node *v*_1_ tends to express at a nominal level when its inhibitor (e.g., node *v*_3_) is actively regulating (i.e., this inactivator's state is expressed below its threshold of action). In the more complex case of node *v*_2_ involving both the upstream activator node *v*_1_, and suppressor node *v*_4_, we have *K*_1_({1,4}) = 1 indicating that under the combined and opposing regulatory actions of nodes *v*_1_ and *v*_4_, node *v*_2_ will evolve toward a nominal state of 1 as dictated by its image Y^t^. Typically, the set of logical K values are learned from experimental time course and steady state measurements. A more detailed description of these parameters is given in section Simplification With ROMDDs. Formally, the transition state image of each entity *y*_*i*_ at time *t*, or $y_{i}^{t}$, is defined as:

(1) $$\begin{array}{l} y_{i}^{t}=\sum_{I\subseteq q(i)}K_{i}\left(I\right)[\prod_{j\in I}S^{u_{i\;j}}\left(x_{j}^{t},w_{i\;j}\right)\\ \;\;\times \prod_{j\in q(I)\backslash I}\left(1-S^{u_{i\;j}}\left(x_{j}^{t},w_{i\;j}\right)\right)] \end{array}$$

Where *q(i)* is the in-degree set of components *v*_*i*_ (i.e., set of regulators of *v*_*i*_), *I* a subset of *q(i)*, ∏ is the multiplicative operator and ∑ is the additive operator. Parameter *u*_*ij*_ is a Boolean flag indicating the polarity of the incoming edge *e*_*ij*_ and is true when $x_{j}^{t}$ is a promoter. Y^t^ = [$y_{1}^{t}$, …., $y_{N}^{t}$] is called the image vector of the regulatory graph G with N components given its current state vector X^t^ = [$x_{1}^{t}$,…, $x_{N}^{t}$]. $S^{u_{ij}}$ ($x_{j}^{t}$, *w*_*ij*_) is a threshold function that determines whether the expssreion level *x*_*j*_ of node *v*_*j*_ is sufficient to exercise a control action e.g., promote (or suppress) a regulatory target *x*_*i*_:

(2) $$S^{u_{ij}}(x_{j}^{t},w_{ij})\;=1↔\{\begin{array}{c} u_{ij}=1\wedge \left(x_{j}^{t}\geq w_{ij}\right),\\ u_{ij}=0\wedge \left(x_{j}^{t}<w_{ij}\right) \end{array}$$

Where ↔ indicates a logical biconditional equivalence, ∧ a logical conjunction AND, and where *w*_*ij*_ is the interaction threshold of the incoming edge *e*_*ij*_ where it takes a value within [1, *l*_*i*_]. Expression level *l*_*i*_ is the maximum state level that entity *v*_*i*_ might assume. It can be shown (Devloo et al., 2003) that Eqation (1) reduces to *K*_*i*_*(I*_*a*_*)* where:

(3) $$I_{a}:=\left\{i\in V\;|(i,j)\in E⋀S^{u_{i\;j}}\left(x_{j}^{t},w_{i\;j}\right)\right\}$$

Where *V, E*, and *I*_*a*_ are the set of all entities, all edges in the network and active interactions on an entity *v*_*i*_, respectively. Conventional operators : = and ∈ signify a defining equivalence and element set membership, respectively. Therefore, the set of all active interactions on a node is in fact denoted in each case by a unique *K*_*i*_*(I*_*a*_*)* logical value that collectively defines the image of that node (see Figure 1A). The state of the network at the next time point (X^t+1^) is determined by choosing an updating scheme such as synchronous or asynchronous (Sedghamiz et al., 2018). Under the synchronous schedule, all of the entities in vector X^t^ change their expression levels toward Y^t^ simultaneously, while under the asynchronous time update only a single entity is allowed to change its expression level at any given time. We have also reported an alternative method involving priority updating which more readily captures different activation timescales such as those that might exist across levels of biology and physiological compartments (Sedghamiz et al., 2017, 2018).

### Preprocessing

Our framework consists of two preprocessing stages; *function simplification with ROMDDs* and *one-hot encoding*. The former employs ROMDDs to represent a function describing entity *v*_*i*_ in its simplest possible form. The latter converts a multi-valued network into an equivalent Boolean model.

#### Simplification With ROMDDs

Intuitively, each *K*_*i*_*(I*_*a*_*)* is a propositional formula consisting of one or more literals. The disjunction of *K*_*i*_*(I*_*a*_*)* defines the state level image (e.g., $y_{i}^{t}$) of an entity (Sedghamiz et al., 2018). For instance, the equivalent propositional formula for *v*_4_ in Figure 1A in an unsimplified form might be written as;

(4) $$y_{4}^{t}=\{\begin{array}{c} 0↔K_{4}\left(\phi \right):=\left[\left\{\left(x_{3}^{t}=0)\vee (x_{3}^{t}=1\right)\right\}\wedge \left\{\left(x_{4}^{t}=0)\vee (x_{4}^{t}=1\right)\right\}\right]\\ 1↔K_{4}\left(\left\{3\right\}\right):=\left[(x_{3}^{t}=2)\wedge \left\{\left(x_{4}^{t}=0)\vee (x_{4}^{t}=1\right)\right\}\right]\\ 2↔K_{4}\left(\left\{4\right\}\right):=\left[(x_{4}^{t}=2)\wedge \left\{\left(x_{3}^{t}=0)\vee (x_{3}^{t}=1\right)\right\}\right] \end{array}$$

Therefore, each entity *v*_*i*_ requires $2^{q_{i}}$, *K*_*i*_*(I*_*a*_*)* parameters to be fully defined; where *q*_*i*_ is the number of inputs to *v*_*i*_ (indegrees). Here again, : = signifies a defining equivalence, ↔ a logical biconditional equivalence, ∧ a logical conjunction AND, and ∨ a logical disjunction OR. The number of literals in a function associated with *v*_*i*_ grows exponentially as its number of inputs or the size of fan-in increases. Thankfully, there exist logic synthesis algorithms developed to perform the similar task of reducing the number of components during the design of an electrical circuit (Sentovich et al., 1992). These algorithms mostly rely on generalization of Reduced Ordered Binary Decision Diagrams (ROBDDs) to ROMDDs. In this study, we employ the logic synthesis algorithm introduced in work by Mishchenko and Brayton (2002). For instance, applying this simplification to Eq. 4 would result in a reduction of the number of literals in the last condition (where $y_{4}^{t}$ = 2) from 5 to 1:

(5) $$\left(y_{4}^{t}=2)\;↔(x_{4}^{t}=2\right)$$

#### One-Hot Encoding

One of the most commonly employed methods to deal with multi-valued variables in logic synthesis is one-hot encoding. For example, if the state of entity *v*_*i*_ is ternary (e.g., *x*_*i*_ = {0,1,2} or {low, medium, high}), it might be represented by a three-bit vector *x*_*i*_ = [*x*_*i*1_*, x*_*i*2_*, x*_*i*3_]. Therefore, if for instance, the first bit of this vector is *true* [i.e., (*x*_*i*1_ ↔ 1)], then *x*_*i*_ = 0. Note that for each multi-valued variable *v*_*i*_ a “don't care” logic expression should be considered as well. For example, if *v*_*i*_ is ternary, then this logic expression is defined as;

(6) $$\left[x_{i1}x_{i2}+x_{i1}x_{i3}+x_{i2}x_{i3}\right]=0$$

This expression states that variable *v*_*i*_ cannot have two states at the same time [e.g., take low and medium (*x*_*i*1_ *x*_*i*2_)]. While other more compact encodings exist such as the one proposed by Didier et al. (2011), we decided in this work to employ one-hot encoding due to its simplicity and convenience of expressing “don't care” or inadmissible states.

#### Intervention Sets

The state of a network with *N* entities at time t is denoted by a vector X^t^ that represents the expression state of each entity at that time. Eventually, the state of a dynamically stable network will over time relax into an attractor where the expression levels of all entities stabilize. An attractor contains a set of states such that once a network reaches any of them, it will transition among those states indefinitely. An attractor with only a single state is called a steady state. It has been proposed that cell differentiation toward distinct and stable cellular phenotypes may correspond to a migration into separate attractors of a different type, shape and location in the state space (Thomas and Kaufman, 2001a,b; Chaouiya et al., 2003; Mendoza and Xenarios, 2006). Therefore, it is of utmost interest to study how an attractor might be efficiently and robustly mapped onto another. Finding the minimal number of interventions (e.g., knock-out or knock-ins) in a network that might achieve this goal (e.g., migrate from a steady state to another or enforce an attractor) translates into finding the most influential entities in the network that could be leveraged as effective drug targets (Samaga et al., 2010). The enumeration of MIS is computationally exponential where for a network with *N* entities a set of 3^n^ candidate combinations exists (where *n* ≤ *N*), since each entity might be pharmacologically knocked-out (i.e., −1), over-expressed (i.e., +1), or not modulated at all (i.e., 0). In this study, for a multi-valued state variable *x*_*i*_ = {0, …, *l*_*i*_}, we also assume that a knock-out or knock-in of the corresponding entity would mean that this state variable is maintained constant at a state of 0 or *l*_*i*_, respectively.

#### MIS Computation

In order to compute the MIS, first we need to define:

- A perturbation vector *P* = [*p*_1_*, …, p*_*n*_] for *p*_*i*_ ϵ {-1, 0, 1}; where {-1, 0, 1} stands for *knock-out, no-intervention* and *knock-in*, respectively.
- A goal steady state $G\subseteq X^{t}(P)$ for which a set of variables assume a *desired* target state and where X^t^(P) is the state of the network at time t being acted upon by perturbation P.
- A cardinality C for the number of externally stabilized nodes targeted by the perturbation vector P which is defined as $C=\overset{n}{\underset{i=1}{\sum }}|p_{i}|$; where |.| is the absolute value operator.
- An initial state X^0^(P) : = X_initial_ of the network for which the MIS solutions would be computed.
- A path length *m*; where *t* = [0, *m*] for which under the influence of P the system migrates fully from its initial state to its target or goal state G.

Then, feasible intervention sets are those configurations for which;

(7) $$\{\begin{array}{c} X^{0}\left(P\right):=X_{initial}\;\wedge \left\{\left(\forall p_{i}=1↔x_{i}^{0}=l_{i})\wedge (\forall p_{i}=-1↔x_{i}^{0}=0\right)\right\}\wedge \\ X^{t}\left(P\right):=X^{t+1}(P)\;\wedge \left\{\left(\forall p_{i}=1↔x_{i}^{t}=l_{i})\wedge (\forall p_{i}=-1↔x_{i}^{t}=0\right)\right\}\wedge \\ G\subseteq X^{t}(P)\wedge C\leq c^{\max} \end{array}$$

Intuitively, given an initial state of the network X_initial_, an intervention set is an assignment of P for which the network is steady (X^t^ (P) = X^t+1^ (P)) at the desired goal G. For practical reasons, we assume that there is an upper-bound constraint *c*^*max*^ on the number of entities that might be externally modulated. Furthermore, each intervention set is associated with a path length *m* allowing transition of the system from an initial state to a target or goal state. This might be interpreted as the number of discrete time steps it might take for an intervention to take effect and may therefore be another important parameter to consider. Given this framework, the computation of MIS consists of a series of repeated simulations where the network is initialized at a specific illness start state, or more generally at a random state, and allowed to evolve iteratively until either the current state of the network is identical to its next state (a steady state has been reached) or the maximum number of the allowable steps (path length m) has been reached. During these repeated simulations, all possible combinations of the different candidate perturbations (P vector) are applied at the initial state and maintained constant. For instance, in order to verify if the knock-out of the first entity in the network constitutes a valid MIS, the state of this entity is initialized and maintained at 0 and the temporal evolution of the network is computed within pathlength *m* to see whether the system will settle at the desired goal steady state G. The choice of combination of candidate intervention nodes and the order in which they are assessed is articulated here as a constraintsatisfaction problem which can be efficiently solved by resolving contradictions within the space of constraints.

#### Kleene's Logic

In a conventional two-valued Boolean logic, a network might support none, one or several steady states. Moreover, a network might support cyclic attractors which make the identification of intervention sets based on the conditions in Equation (7) difficult. Samaga et al. (2010) proposed to extend the two-valued Boolean logic to three-valued Kleene's logic. Under Kleene's logic, a logical value U is introduced which extends the two-valued logic into {*false* : = 0; U: = 1; *true* : = 2}. Under this formalism a biological network would always have a single steady state since, for the cyclic attractors and the entities that their stable state might depend on, the initial values can be captured. In other words, under Kleene's logic, the state variable of an entity is either fixed (e.g., ϵ{0, 2}) or unknown (e.g., 1). For instance, based on the conventional Boolean logic, a model might have two attractors whereby in one attractor an entity *v*_*i*_ takes a state value of *On* (i.e., 1) and in the other attractor *Off* (i.e., 0). Under Kleene's logic these two attractors are merged into one where the entity *v*_*i*_ takes an unknown state value instead. Abramovici et al. (1990) employed Kleene's logic for circuit verification and showed its usefulness in failure mode detection in electrical circuits. In this three-valued formalism, the extension of logical operators is straightforward:

(8) $$\{\begin{array}{c} OR\left(A,B\right)\equiv \;Max\left(A,B\right),\\ AND\left(A,B\right)\equiv \;Min\left(A,B\right),\\ \neg (A)\equiv \left[\left\{\neg (A=0)↔2\right\},\left\{\neg (A=2)↔0\right\},\left\{\neg (A=1)↔1\right\}\right] \end{array}$$

Where ↔ signifies logical equivalence, ¬ signifies logical negation (NOT), and ≡ defines an identity. Now, it is even possible to set the initial state X_initial_ of the network to be completely unknown, that is where all the entities take a value of *x*_*i*_ = 1.

#### Stochasticity and Time Updates

**Figure 2:**
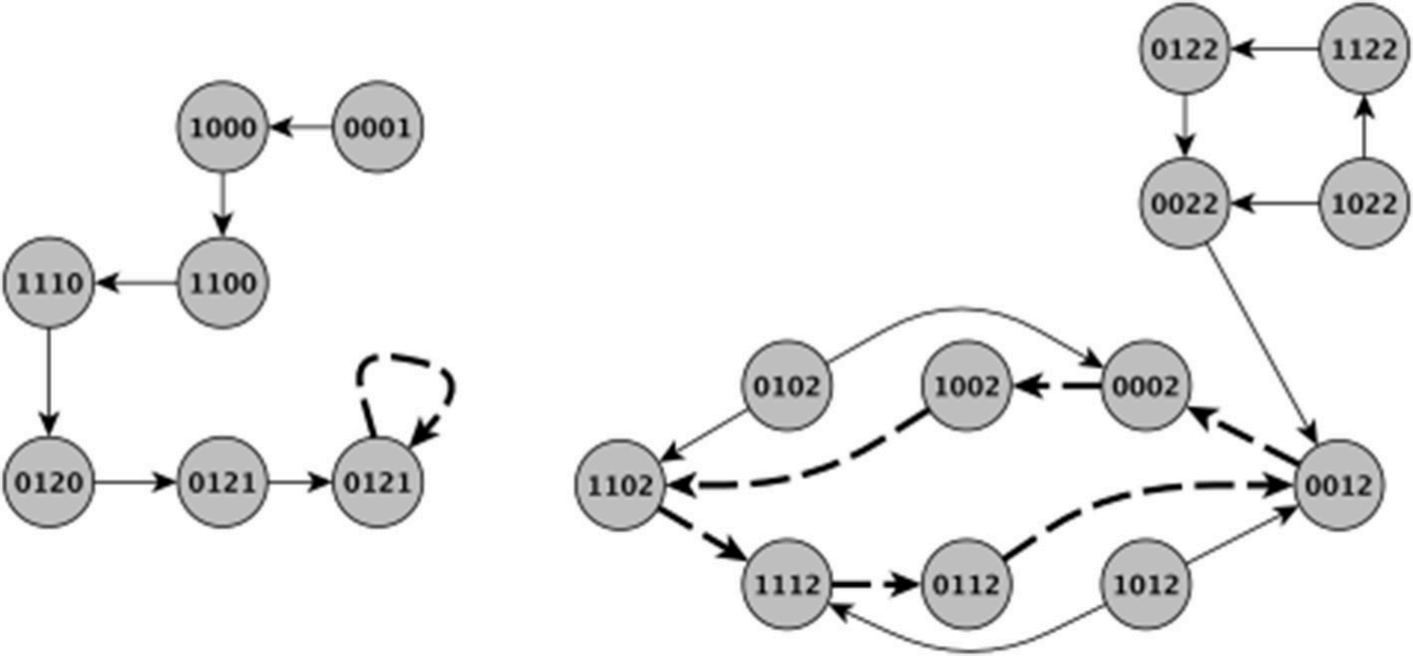
Visual comparison of synchronous and asynchronous time updates. The thick dashed edges indicate a stable attractor. Note that the basins of attraction are different between the update schemes and therefore different initial states might result in different type of stable behavior. For example, in the left panel the system evolves from an initial state of (0001) under synchronous updating to eventually a stationary steady state of (0021). In the right panel however, starting from either (1122) and (1022) eventually leads an oscillatory behavior around a cyclic attractor. Contrary to asynchronous updating, when applying synchronous updating each state has a single successor making the set of states leading to an attractor very different. It is this multiplicity of paths afforded by asynchronous updating as well as the added effect of noise that motivated the Monte Carlo simulations in this work. The indices of the state variables are the same as in Figure 1.

As mentioned earlier, there are two common update schemes typically employed in logical modeling, namely *synchronous* and *asynchronous* updating (Sedghamiz et al., 2017). Asynchronous update implicitly considers the stochasticity associated with time-delay of entities in the network while on the other hand, synchronous update is computationally more efficient since each state in a network's State Transition Graph (STG) has a unique successor (Chaouiya et al., 2003). Figure 2 graphically illustrates both updating schemes applied to the basic model of HPA axis regulation of Figure 1A. While the geometry of the basins of attraction obtained under each scheme can vary, the steady states, specifically the stationary points, are the same. To take advantage of this computational efficiency, we have chosen here to incorporate biological stochasticity by combining synchronous update with a stochastic Monte Carlo simulation scheme introduced in earlier work by our group (Sedghamiz et al., 2018). In this approach, a probability of failure ∈ ϵ [0, 1] is introduced for which an entity might disobey its defined logical function. First, we compute the list of interventions based on the synchronous assumption. Then, for each intervention, we run parallel Monte-Carlo simulations for *M* times (e.g., 1,000) by assuming a chance of failure in each entity's regulatory function (e.g., ϵ = 0.05). The number of times a given intervention successfully reaches its goal destination corresponds to its robustness. Simulation results produced under ideal conditions (e.g., ϵ = 0 or zero noise) are included in File S1 demonstrating that for each benchmark problem presented here, application of the corresponding set of tabulated MIS prompts a migration of the model system to a final stable state that corresponds exactly to the desired goal state.

#### MIS Ranking

In practice there are several criteria that must be considered when designing an intervention. Recall that in this work we conceptualize treatment efficacy as an aggregate of efficiency in response and robustness of outcome. We apply these and the following other factors to rank the potential feasibility of each predicted intervention set:

- *Cardinality*: Number of intervened entities (C). This might be interpreted as the complexity of the intervention.
- *Efficiency*: Number of transitions required to achieve a goal under that intervention (*m*).
- Off-target Effects: Number of entities that are over-expressed or down-regulated but were not included in the goal steady state G.
- *Robustness*: Number of times that an intervention is successful under Monte-Carlo simulations normalized by the number of runs.
- *Safety*: This is interpreted as the set of entities that are not permitted to be targeted directly nor effected by intervention downstream of a target. Safety is not a feature employed in our ranking procedure but rather a hard constraint provided by the user in the form of a set of entities in the network.

It is important to remember that in this formulation the goal steady state G is articulated as a rigid constraint. Though some MIS will rank more favorably than others based on these criteria, all candidate MIS must deliver complete and exact adherence to this goal steady state or they will not be retained in the solution set.

## Implementation

**Figure 3:**
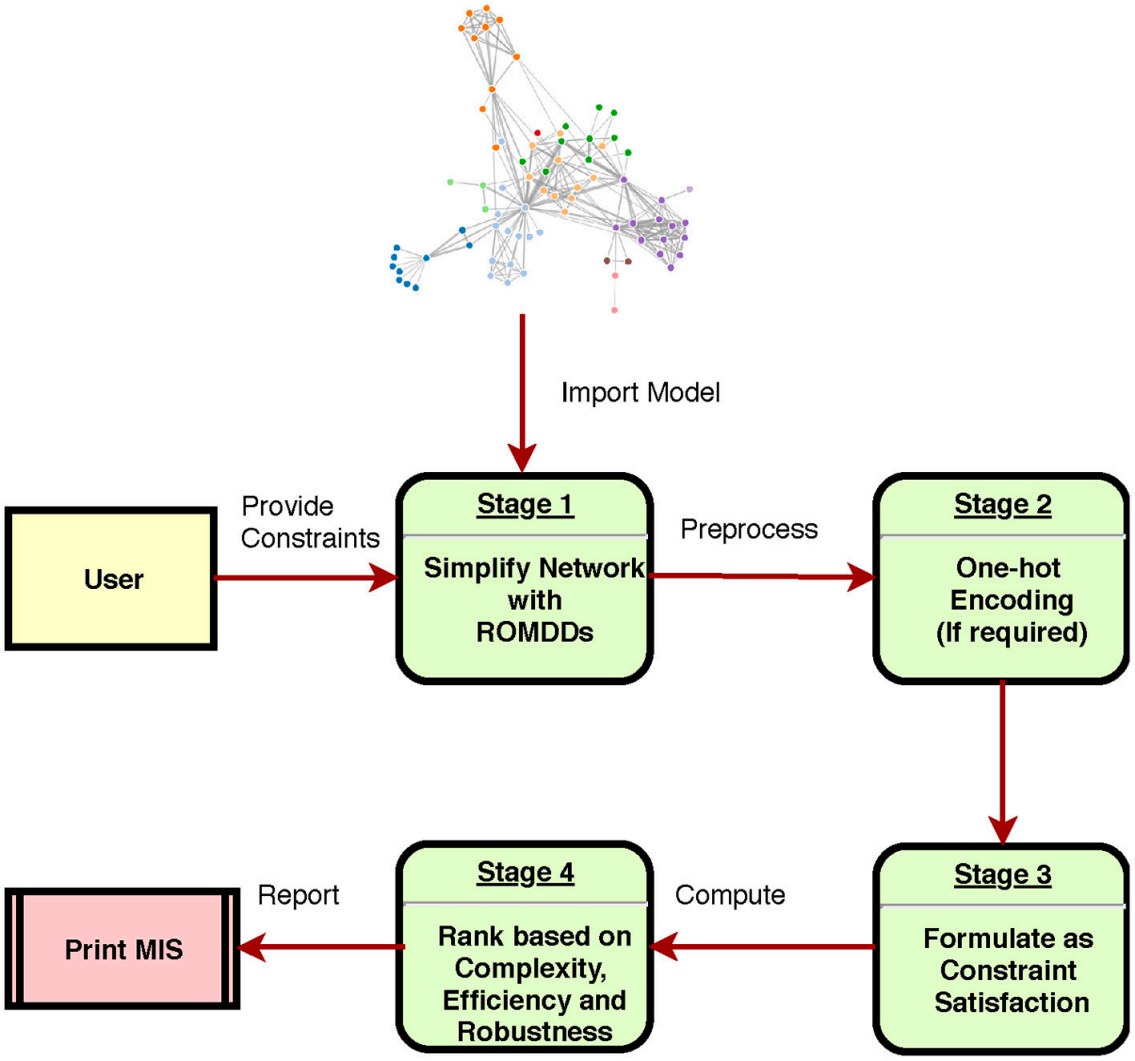
Different stages of computing the intervention sets in a regulatory network.

**Figure 4:**
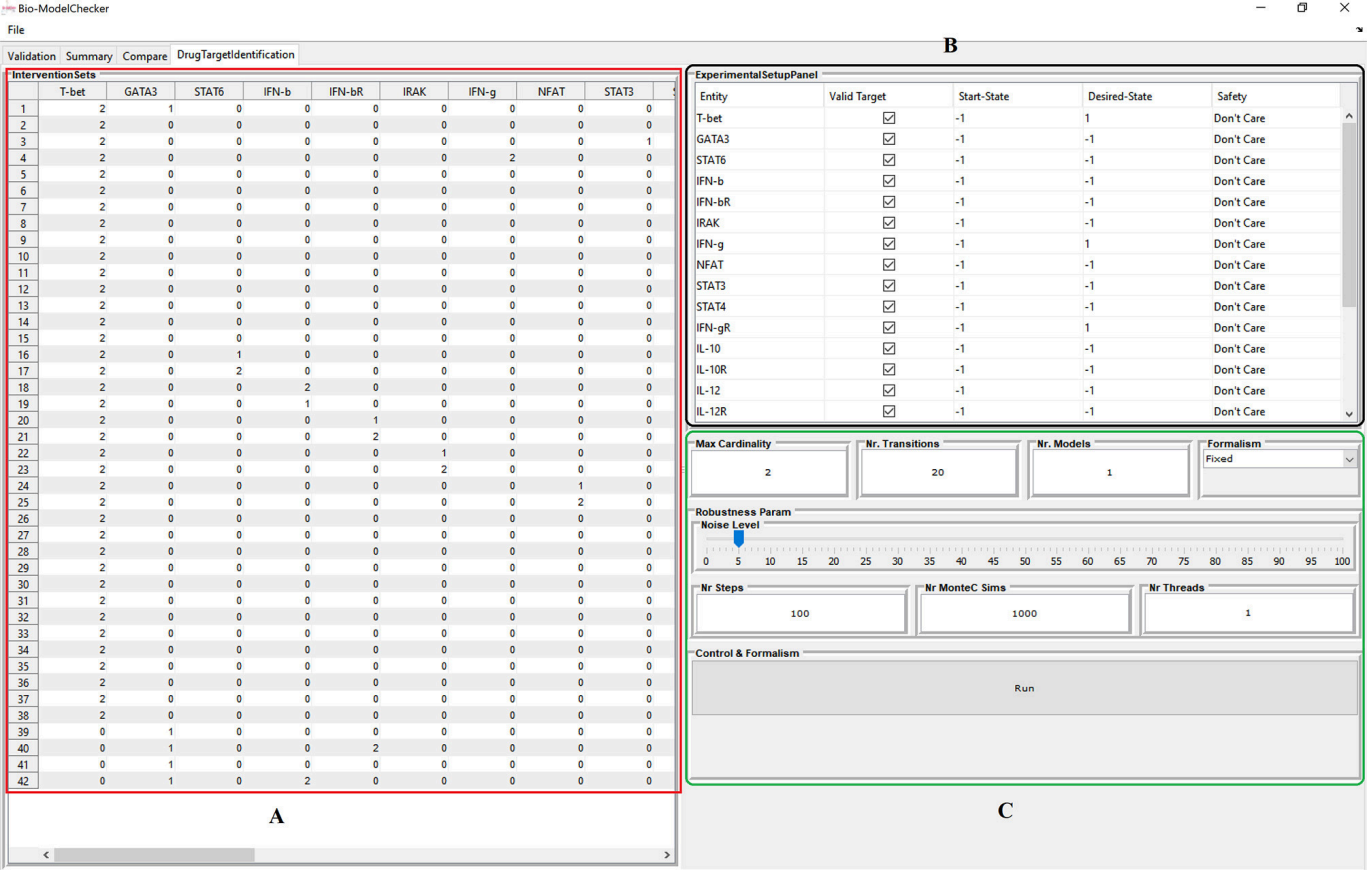
Snapshot of the BioModelChecker software virtual *in-silico* lab for identification of intervention sets. **(A)** Computed interventions. **(B)** Experimental design panel that allows the user to setup the constraints and requirements. **(C)** Options for computation of robustness based on Monte-Carlo simulations.

The proposed framework is implemented in BioModelChecker (BioMC), a standalone software developed by our group for the reverse engineering and analysis of regulatory networks based on Constraint Satisfaction (CS) techniques (https://github.com/hooman650/BioModelChecker). BioMC first translates the problem to a CS framework and then solves it with the state-of-the-art solvers such as *Google's Operations Research tools (OR-tools)* (Perron, 2011) and *Chuffed* (Chu et al., 2014) that rely on Lazy Clause Generation (LCG) techniques (see Sedghamiz et al., 2017 for more details). All the benchmarks employed in this study were first parameterized with BioMC. The top performing models based on the ranking criteria proposed in our earlier work (Sedghamiz et al., 2017) were selected for MIS analysis. The complete work-flow is illustrated in Figure 3 and a snapshot of the BioMC *in-silico* lab in Figure 4. The T-Helper benchmark and its corresponding logical transition functions is adapted from Mendoza and Xenarios (2006). BioMC is also extended to support the fixed Boolean logic rules proposed by Mendoza and Xenarios (2006) and ultimately only requires the modeler to provide topology of the network. All of the benchmarks along with their discretized experimental data are accompanied with BioMC. Furthermore, the regulatory interactions used to define each example network presented here are listed in File S2.

## Results

### HPA Axis

The Hypothalamic-Pituitary-Adrenal (HPA) axis is one of the most fundamental components of the body in regulating the response to stress. Due to its important regulatory role, it is no surprise that the HPA axis has been associated with a number of complex chronic diseases such as Gulf War Illness (GWI) and Myalgic Encephalomyelitis/Chronic Fatigue Syndrome (Beishuizen and Thijs, 2004; Morris et al., 2017). In our earlier work (Sedghamiz et al., 2018), we developed a discrete multi-valued model of the HPA (Figure 1A) that supports two cyclic attractors (Figure 1B) and demonstrated the predicted effects of administering an antagonist of the glucocorticoid receptor R. In the present work, we formally compute all feasible intervention sets that drive this network away from a hypocortisolic state toward higher levels of cortisol expression (Cort). Figure 1C describes all 17 intervention sets where at most 2 targets are modulated that force the network into an attractor where cortisol is expressed at mid-range to high levels by this model. Predictions suggest that cortisol supplementation, applied alone or in combination with the modulation of other targets, offers maximal robustness. However, direct modulation of glucocorticoid levels with for example prednisone present with increased risks of adverse events associated with broad immunosuppression and metabolic upset (Dineen et al., 2018; Graziadio et al., 2018). Recent pharmacological advances are making this approach more feasible but these remain at the stage of phase I clinical trials (Hoffman et al., 2018). Alternatively, MIS3 and MIS13 are both robust and efficient and only require either ACTH or glucocorticoid receptors (R) to be perturbed. Inhibition of R has already been shown to be an effective and common procedure for restoring appropriate cortisol levels (Clark, 2008) and accordingly was identified as one of the most promising intervention strategies using our MIS approach.

### T-Helper GRN

**Figure 5:**
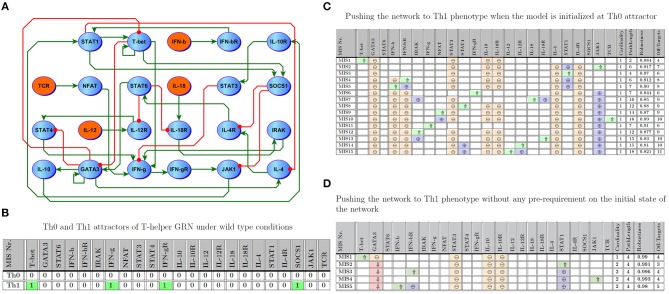
T-Helper Network. **(A)** Regulatory interactions involved in the model; the network consists of 23 entities where 4 are inputs. **(B)** Attractors describing Th0 and Th1. **(C)** Minimal number of perturbations required to enforce Th1 when the network is initialized at Th0; ⇑ (light green), ⇓ (light red), ⊕ (purple), and ⊖ (orange) indicate knock-in, knockout, off-target up-regulated, and off-target down-regulated, respectively. Path length and robustness are computed based on 1,000 Monte-Carlo simulations under ϵ = 0:05 of noise. **(D)** Minimal Intervention Sets to force Th1 without any pre-requirement on the initial state of the network.

As a further validation of this approach, we analyzed a second larger benchmark problem, namely a well-studied and documented immune signaling network describing the differentiation of naive T helper (Th0) cells to either Th1 or Th2 phenotype. The network consists of 23 entities connected by 35 regulatory interactions. This architecture offers a reasonably large number of entities but with sparsely connected interactions (approximately 7% connection density). A detailed description of the dynamics of this model can also be found in Mendoza and Xenarios (2006) and Garg et al. (2007, 2008). Excessive Th1 activation is a common feature in many auto-immune illnesses, while an immune profile supporting over-activation of the Th2 axis has been associated with several forms of allergy (Murphy and Reiner, 2002; Garg et al., 2008). We focus here on the transition from a naive Th0 phenotype to a stable Th1 cell fate. Figures 5A,B illustrate the T-helper differentiation network, as well as the marker expression profiles corresponding to the Th0 and Th1 cell phenotypes, respectively.

Similar to work by Garg et al. (2013), we computed intervention sets with a maximum cardinality of 2 which favor the Th1 steady state. Figure 5C shows 15 MIS solutions where a network initialized at the Th0 naive phenotype would be expected to transition to a Th1 phenotype. In an extension of this framework, Figure 5D illustrates interventions predicted to drive the network into the Th1 attractor regardless of the initial state of the model, extending the problem of differentiation to a broader range of phenotypes representative of documented T cell plasticity (Hirahara et al., 2013; Caza and Landas, 2015). Of note, our predictions show that this more broadly induced plasticity that is independent of initial phenotype (Figure 5D) usually requires direct perturbation of multiple targets, 270 including the master T cell regulators T-bet or Gata3, which are known to be difficult to target directly (Weigmann and Neurath, 2002; Usui et al., 2003). Indeed, of the 5 MIS solutions identified that were independent of initial state, only one single-target strategy emerged, namely manipulation of T-bet. It stands to reason that induction of Th1 from any theoretically achievable state in the network would require a more complex intervention set. Nonetheless, the MIS shown in Figure 5D typically exhibit very high robustness and result in fewer downstream off-target markers being indirectly perturbed, a potential byproduct of jointly manipulating two targets. In comparison, those interventions computed based on a specific initial state of the network (e.g., Th1 differentiation from naive Th0 as depicted in Figure 5C) consisted mostly of one intervention target. For instance, MIS6 and MIS11 in Figure 5C indicate that promoting over-expression of IFNγ or activation of its receptor IFNγR, both characteristic markers of the Th1 phenotype (Figure 5B), would prompt a migration from Th0 to Th1.

Interestingly, stimulation of another characteristic marker of Th1 fate, SOCS1, was not predicted to be sufficient to induce polarization to this phenotype. This exemplifies the point that direct manipulation of differentially expressed markers in absence of a deeper knowledge of network structure may or may not yield the desired effect. High robustness (0.97 and 0.91) and short state transition path-length (7 for both) nonetheless suggest in this case that these interventions involving IFNγ and its receptor appear especially noteworthy (Garg et al., 2013). Showing slightly lower robustness and efficiency, the remaining MIS candidates identified by these simulations include known drivers of Th1 differentiation such as IL-12, IFNγ and T-bet. Moreover, every identified MIS supports persistent activation of T-bet and inactivation of GATA3, the hallmark switch in the activation state of these master transcription factors governing Th1/Th2 differentiation. This list of candidate targets can be further refined based on their ease of targeting or the availability of specific drugs. While T-bet activation is predicted to be a highly robust means of inducing Th1 differentiation in both the naive Th0 (Figure 5C) and in the context of a more broadly induced plasticity (Figure 5D), it is known to be difficult to target directly by pharmacological means. In such cases, a different target such as STAT1, for which pharmacological activators are available (Lynch et al., 2007), might be selected instead. Importantly, the optimal targets need not necessarily be constitutively expressed in the target state: in this example, transient STAT1 activation is predicted to induce Th1 differentiation even though STAT1 is not constitutively active in Th1 cells. It is important to note that these target states constitute self-sustaining resting states and that once reached these interventions may be discontinued without compromising remission.

### Vaccine Response Network

In this third example, we apply our approach to a network consisting of a somewhat smaller number of entities compared to the previous T helper network, but where these entities are much more extensively interconnected. Our research group has identified a pediatric population, comprising some 10% of children that respond poorly to recommended routine vaccinations in their first year of life, developing sub-protective antibody responses to two-thirds or more of the immunizations given. These children correspond to a clinical phenotype we have defined as “low vaccine responders (LVR),” as opposed to “normal vaccine responders (NVR)” (Pichichero et al., 2016). We have reported defects in immune cell function among LVR children, including impaired polyclonal T cell response (Pichichero et al., 2016) and reduced innate immune activation by Toll-like receptor stimulants (Surendran et al., 2016, 2017).

**Figure 6:**
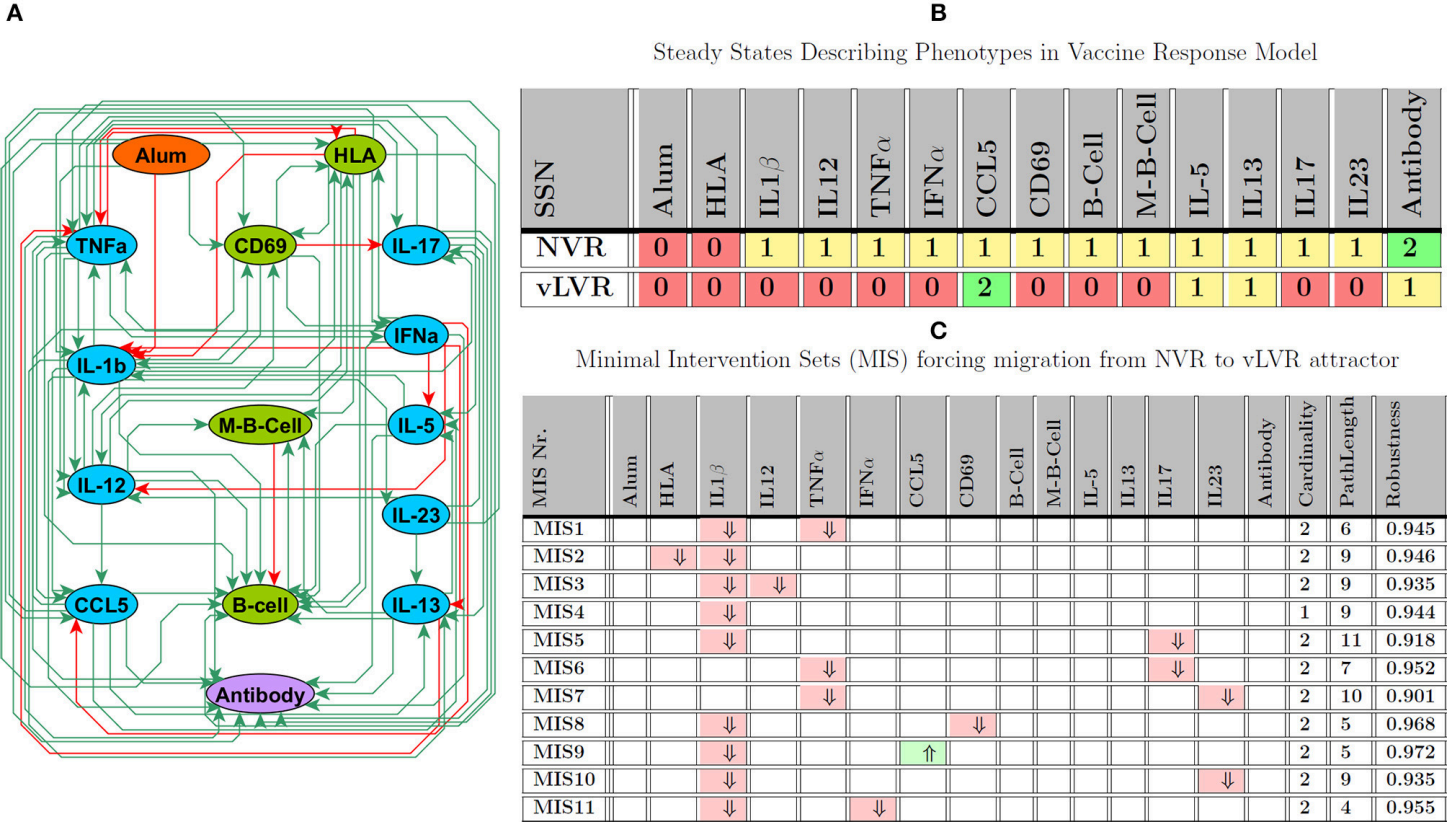
Vaccine Response Network (VRN). **(A)** Regulatory interactions describing the VRN network derived from literature and experimental knowledge; note that, in this analysis Alum represents vaccine adjuvant, while the specific antigen is modeled as stimulating HLA. **(B)** Attractors describing NVR and LVR phenotype; 0, 1, 2 intuitively indicate low, medium, and high expression levels. **(C)** Minimal number of perturbations required to force LVR based on the assumption that the model starts at NVR state; ⇑ (light green), ⇓ (light red), indicate knock-in and knockout, respectively. Path length and robustness are computed based on 1,000 Monte-Carlo simulations under ϵ = 0:05 of noise.

To further the study of this population, we assembled a preliminary Vaccine Response Network model, depicted in Figure 6A, that consists of 15 entities linked by 81 regulatory interactions which translates into an approximate connection density of 36%. This is typical of immune cell signaling systems (Frankenstein et al., 2006). These regulatory interactions were extracted broadly from the scientific literature by applying the MedScan natural language processing engine in a naive context agnostic text mining of the Pathway Studio database (Elsevier, Amsterdam) which is comprised of over 4 million abstracts and full text references (Novichkova et al., 2003) encompassing *in vitro, in vivo* animal as well as human studies. This first coarse-grained regulatory model focuses primarily on mechanisms important in the generation and maintenance of protective vaccine immunity in the periphery, aggregating additional phenomena occurring collectively across the nasopharyngeal, peripheral blood and lymphatic circulatory compartments. As such the model offers a first glimpse of potential immune disequilibrium in LVR useful in identifying gaps in our understanding and corresponding experimental strategies by which the latter could be addressed. In essence, these MIS offer a therapeutically-motivated validation strategy.

Vaccination was modeled as an exogenous activation of HLA by vaccine antigen and stimulation of immune response with Alum as an adjuvant. In preliminary simulations informed by our published descriptions of LVR and NVR vaccine responses, our multi-valued logical model supported two attractors exhibiting immune response profiles that might be interpreted as LVR and NVR (see Figure 6B). In Figure 6C, we explore avenues supporting the onset of LVR by identifying perturbation sets that induce a migration from NVR to this persistent phenotype, highlighting potential insults and response mechanisms that might underlie the etiology of LVR. Results of this analysis suggested that onset was not a single-point failure and that concurrent upset of at least 2 immune mediators was generally required to promote deficits in vaccine response. Among the MIS solutions identified for inducing LVR, almost all required the suppression of IL-1β, a central regulator of inflammation (Dinarello and van der Meer, 2013). While IL-1β suppression alone was predicted to be sufficient to induce migration to the LVR attractor, the robustness of this intervention could be increased by simultaneous perturbation of additional network nodes. For example, co-occurrence of reduced IL-1β expression with over-expression of CCL5 is predicted as the most robust or inescapable path of LVR onset. The next most common targets were TNFα, another central regulator of innate immunity, followed by IL-17 and IL-23, both involved in Th17 differentiation and function (Toussirot, 2012). Indeed, deficiency of these in combination with each other or paired individually with low IL-1β expression also supported robust vulnerability to LVR. This suggests that simultaneous disruption of innate inflammatory responses and T cell activation may be a major cause of sub-protective vaccine responses. Although most cytokines are differentially regulated in the NVR and LVR states, the only single target sufficient to induce LVR was IL-1β.

It is important to note that unlike the previous examples which consist of established benchmark problems, this last circuit remains a first exploratory model of peripheral immune function in low vaccine response children. The experimental data employed were steady state measurements of the model and are accompanied with BioMC. Indeed, while unique models were used to derive MIS for HPA axis function and T helper polarization, the complexity of the vaccine response circuit was such that 132 variants of this model could explain the limited experimental data available during the parameterization, since the problem was under-determined due to the limited amount of experimental data available. In this particular case, in addition to other criteria defined by our group (Sedghamiz et al., 2017) for ranking the feasible models, the clinical experience of a domain expert was used to help select the most immediately plausible model to serve as a basis for estimating the MIS in Figure 6C. As new data becomes available, for example as might result from experimental assessment of MIS in Figure 6C, one may expect such variants to eventually converge toward a single consensus model.

## Discussion and Conclusion

In this study, we propose an efficient CS based formalism for the computation of minimal intervention sets consisting of parsimonious groups of targets in a biological regulatory network that if concurrently promoted or inhibited would disrupt one homeostatic regime in favor of another more desirable regulatory equilibrium. The enumeration of these sets is computationally exponential as we represent the dynamic behavior of these biological networks with the highest biologically relevant fidelity using our group's refinement of a multi-state logic (Sedghamiz et al., 2017, 2018) originally proposed by Thomas et al. (1995), Thomas and Kaufman (2001b), and Chaouiya et al. (2003). We address this complexity by first applying efficient logic synthesis techniques developed in the microelectronics community to simplify the logical equations that describe the state change dynamics of each entity in the network. We then generalize these using one-hot coding to support the multi-valued logic required to adequately represent biological mediators that express and act over a broader, more continuous biological entities (e.g., signaling proteins). The combination of network simplification techniques along with Constraint Satisfaction enabled us to analyze relatively larger models. For instance, the analysis of LVR network which has 15 ternary nodes or an equivalent of 45 binary entities has a state transition graph size of 2^45^ ≈ 35 × 10^12^ that must be traversed for each of candidate combinations ${\left[\sum_{i=0}^{C}\left(\begin{array}{c} N\\ i \end{array}\right)\right]}^{2}=14641$ for maximum cardinality of C=2 and a network of *N* = 15 entities, where $\left(\begin{array}{c} n\\ k \end{array}\right)$ is the binomial coefficient). Our proposed method found all of the intervention sets for this model within a maximum cardinality of 2 in <2 min on an Intel core i7 machine. Contrary to conventional evolutionary based optimization methods, our proposed framework exhaustively explores the whole search space and finds *all* of the MIS candidates. This is very important aspect to consider, since as indicated in several of this study benchmarks, some MIS candidates might be equally feasible and can only be ruled out during the chemical compound library search. The selection of target mediators into these intervention sets is directed at improving overall performance measures such as expediency of treatment response and the reliability of the outcome reported previously by our group as response efficiency and robustness, respectively (Sedghamiz et al., 2017). We extend the concurrent performance objectives in this context to also promote minimal invasiveness which we articulate as a minimization of the number of intervention targets directly modulated (set cardinality) as well as the targets indirectly mediated by virtue of regulatory actions propagated downstream, captured here under the additional metric labeled safety. Importantly, while these targets are first identified under essentially ideal noise-free conditions using the less onerous synchronous update of the network's state, they are then tested extensively and these metrics recomputed under increasing levels of noise to simulate variations in decisional timing and regulatory outcome. Finally, intervention solutions are computed that are specific to a given initial state, extending the classical problem to suit a precision medicine environment.

In this work, we first demonstrate and test this approach by computing intervention sets for two well-established benchmark problems consisting of regulatory networks existing at the organ system (HPA axis) and cell signaling levels of biology (T helper cell fate selection). In both cases, known and accepted intervention targets are consistently recovered and assigned a high overall performance based on the metrics described here. In the case of the HPA stress response axis, antagonism of glucocorticoid receptors is a well-accepted means of re-establishing cortisol levels. The importance of considering context when designing combination therapy in a regulated system is demonstrated even with this simple network where for the same target an agonist or an antagonist may be used depending on the choice of companion target suggesting a context-specific directionality in joint interventions. Indeed, in the context of therapeutic increases of either CRH, ACTH or cortisol, both an agonist or an antagonist of glucocorticoid receptor R will achieve the same desired result, both equally disrupting the self-perpetuating cycle of chronically low cortisol levels. Joint modulation of R is required but is independent of direction. This is only true in the context of a combination therapy, as when modulated alone, receptor activity must be antagonized.

Another interesting observation also derives directly from the networked architecture of these systems and further challenges the conventional approach to therapy of attempting to individually adjust markers to their desired “normal” state. While in the case of the HPA axis and T helper polarization direct manipulation of some of the markers will favor migration of the system to a target equilibrium state, this does not apply broadly and is the exception rather than the rule. For example, exogenous stimulation of IFNγ which is constitutively up-regulated in the target Th1 phenotype will indeed induce a transition from naive Th0 toTh1. However, exogenous stimulation of SOCS1, also constitutively up-regulated in Th1 cells, will not promote this transition even with the help of a co-factor. Behaviors such as this are becoming increasingly appreciated as an underlying cause treatment resistance to single-target interventions (Hiddingh et al., 2014; Lavi et al., 2014). This being said if we possess a reasonable understanding of the regulatory interactions that control a system, and the current initial state at which we are applying the intervention, it is often possible to judiciously chose a broad-acting or master regulator capable of resetting equilibrium. Indeed 15 single target strategies were identified for prompting Th1 polarization from the Th0 reference state. In comparison, with the sole exception of T-bet, we require a coordinated modulation of at least 2 targets for the induction of Th1 polarization to be robust across a broad range of different initial states. Indeed, biomarker panels have been shown to improve the effectiveness of single target conventional therapies by better defining high-response subgroups, in other words individuals that occupy similar initial or untreated resting states (Capobianco, 2017; Fricker et al., 2017).

These same observations from our analysis of established benchmark problems also emerged when demonstrating the scalability of this framework to a much more densely connected prototype network of immune signaling important to the generation of a protective vaccine response. Inhibited expression of central inflammatory mediators (IL-1β or TNFα), especially in conjunction with type-17 T cell effectors (IL-17 and IL-23), was required to blunt the response to common childhood vaccines. The model was generally resilient to deficiencies (knock down) in individual mediators, with the exception of IL-1β. Indeed, despite the model's simplicity the predicted combinatorial nature of upsets required to increase the risk of persistent illness is consistent with the general resilience of normal regulatory homeostasis and aligns in principle with the reported approximate 10% prevalence of this condition (Pichichero et al., 2016). While these predicted deficits remain based on a preliminary model of peripheral immune signaling, this framework has nonetheless produced a set of testable hypotheses that formally account for the mechanistic regulatory interactions between these mediators. Indeed, applying a network-based approach to further our understanding of vaccine immunology is highly novel, and formally accounting for regulatory dynamics even more broadly so. Though recent work has demonstrated the use of broad-spectrum surveys of immune markers analysis of this data continues to be centered on marker expression or combinatorial co-expression without formal consideration of interactions linking these markers as members of a signaling network (Rechtien et al., 2017).

The specific area of vaccine immunology notwithstanding, the analysis and application of network biology to the identification of drug targets continues to evolve broadly in the study of immunology with cancer immunology driving many of these developments. The bulk of these applications however continue to focus mainly on the analysis of network topology and the distribution of marker-to-marker associations (Wang et al., 2014). So far very few studies of drug target selection consider network dynamics and these rely mainly on conventional pharmaco-kinetic approaches (Joslyn et al., 2018). These conventional rate equation methods typically require large sets of time course data uniformly surveyed across samples. In contrast, the use of constraint-based methodology described here accommodates sparsely sampled and incompletely surveyed data by design. Moreover, it provides significant gains in efficiency compared to mainstream goal-seeking approaches (Craddock et al., 2015) by solving the reverse problem of constraint violations. While the discrete qualitative models interrogated here do not have the same temporal resolution as conventional pharmacokinetic (PK) models they nonetheless rigorously enforce the correct causal sequence of events allowed by the regulatory logic leading the to correct recovery of known therapeutic targets. Moreover, the fidelity of these network dynamics to those expressed by their counterparts in the continuous space can then be controlled as required by setting a maximum number of discrete state levels for each individual entity of interest. Intermediate formulations exist which formally integrate continuous and discrete dynamical models (Heemels et al., 2010) but again these increases in fidelity come with added complexity and limitations in scalability. We submit that the framework proposed here offers an attractive data-efficient alternative to conventional PK-based target identification schemes, while being easily scalable and capable of supporting the complex competing goals of therapy design.

## Author Contributions

HS developed and evaluated the mathematical analysis tools, ran simulations, prepared graphics, and drafted the initial manuscript. MM and MP helped design the biological model and contributed to the biological interpretations in the manuscript. TC contributed to prior work with the model and reviewed the manuscript. DW guided core algorithmic changes leading to significant increases in efficiency and reviewed the manuscript. GB directed the work, contributed directly to the development of the original and revised frameworks, and was a major contributor in writing the manuscript. All authors read and approved the final manuscript.

### Conflict of Interest Statement

The authors declare that the research was conducted in the absence of any commercial or financial relationships that could be construed as a potential conflict of interest.

## Supplementary Material

BioModelChecker (BioMC), a standalone software developed by our group for the reverse engineering and analysis of regulatory networks based on Constraint Satisfaction (CS) techniques is freely available at https://github.com/hooman650/BioModelChecker. Also available at this site are accompanying user documentation and example problems.

File S1 **Supplemental Figures S1–S3**. Migration trajectories of benchmark systems under their respective MIS.

File S2 **Supplemental Tables S1–S3**. Lists of regulatory interactions used in the HPA, T-helper, and LVR example networks.
