# Supplementary material for: Computation of Robust Minimal Intervention Sets in Multi-Valued Biological Regulatory Networks
Source: Front Physiol. 2019 Mar 19;10:241. doi: 10.3389/fphys.2019.00241 (PMC6433979; doi:10.3389/fphys.2019.00241)
Supplement: File S1 — Supplemental Figures S1–S3. Migration trajectories of benchmark systems under their respective MIS. [file Table_1.DOCX]

Supplementary Material

Supplemental Figures S1-S3

Hooman Sedghamiz ^1^, Matthew Morris ^1^, Darrell Whitley ^2^, Travis J. A. Craddock ^3;4^, Michael Pichichero ^5^ and Gordon Broderick ^1;6^*

^1^ Rochester General Hospital Research Institute, Center for Clinical Systems Biology,

Rochester, New York, United States

^2^ School of Computer Science, Colorado State University, Fort Collins, Colorado,

United States

^3^ Departments of Psychology and Neuroscience, Computer Science, and Clinical

Immunology, Nova Southeastern University, Florida, United States

^4^  Clinical Systems Biology Group, Institute for NeuroImmune Medicine, Nova

Southeastern University, Florida, United States

^5^ Rochester General Hospital Research Institute, Center for Infectious Diseases and

Immunology, 1425 Portland Ave, Rochester 14621, New York, United States

^6^ Rochester Institute of Technology, Department of Biomedical Engineering,

Rochester, New York, United States

*** Correspondence:**Gordon Broderick, PhD, Director

Center for Clinical Systems Biology, Rochester General Hospital

1425 Portland Avenue

Rochester, NY 14621
gordon.broderick@rochesterregional.org

**Figure S1. Simulated MIS response of HPA model**. Simulated reversal of persistently depressed cortisol levels (node 3) in the model hypothalamic-pituitary-adrenal (HPA) axis model in **Figure 1A** in response to the application of 17 MIS candidates tabulated in **Figure 1C**. The goal state was defined as a stable upregulation to an expression level of 2 for cortisol (node 3) only. All candidate MIS induce stable activation of cortisol (dashed line with green diamond). The final states for CRH (node 1), ACTH (node 2) and GR (node 4) were not constrained to specific target values and hence vary in final expression from one MIS to another.

**Figure S2. Simulated MIS response of T helper cell model**. Simulated migration of a t helper cell from a naïve state (Th0) to a T helper type 1 (Th1) state based on the cell signaling model of **Figure 5A** in response to the application of 15 MIS candidates tabulated in **Figure 5C**. The goal state was defined as a stable upregulation of T-bet, IFN-γ, IFN-γR and SOCS1 only (**Figure 5B**). All candidate MIS induce the stable activation of these 4 markers (dashed line with green diamond). The final states for remaining 19 state variable nodes were not constrained to specific target values and hence vary in final expression from one MIS to another.

**Figure S3. Simulated MIS response of Low Vaccine Response model**. Simulated migration of an immune signaling network (**Figure 6A**) from a Normal Vaccine Response (NVR) state to a persistent pathological state of very Low Vaccine Response (vLVR) in response to the application of the 11 MIS triggers of onset tabulated in **Figure 6C**. Once again, all candidate MIS triggers result in the stable induction of the target vLVR pathology (dashed line with green diamond). In this example, the goal state was fully defined in all state variables (**Figure 6B**) and as such all state variables reach their target expression levels in direct compliance with the corresponding constraints.
